# Handling healthcare workforce planning with care: where do we stand?

**DOI:** 10.1186/s12960-015-0028-0

**Published:** 2015-05-24

**Authors:** Mário Amorim Lopes, Álvaro Santos Almeida, Bernardo Almada-Lobo

**Affiliations:** 1 INESC TEC, Faculdade de Engenharia, Universidade do Porto, Porto, Portugal; 2 Faculdade de Economia, Universidade do Porto, Porto, Portugal

**Keywords:** Review, Health-care workforce planning, Supply, Demand, Needs, Health policy

## Abstract

**Background:**

Planning the health-care workforce required to meet the health needs of the population, while providing service levels that maximize the outcome and minimize the financial costs, is a complex task. The problem can be described as assessing the right number of people with the right skills in the right place at the right time, to provide the right services to the right people. The literature available on the subject is vast but sparse, with no consensus established on a definite methodology and technique, making it difficult for the analyst or policy maker to adopt the recent developments or for the academic researcher to improve such a critical field.

**Methods:**

We revisited more than 60 years of documented research to better understand the chronological and historical evolution of the area and the methodologies that have stood the test of time. The literature review was conducted in electronic publication databases and focuses on conceptual methodologies rather than techniques.

**Results:**

Four different and widely used approaches were found within the scope of supply and three within demand. We elaborated a map systematizing advantages, limitations and assumptions. Moreover, we provide a list of the data requirements necessary to implement each of the methodologies. We have also identified past and current trends in the field and elaborated a proposal on how to integrate the different methodologies.

**Conclusion:**

Methodologies abound, but there is still no definite approach to address HHR planning. Recent literature suggests that an integrated approach is the way to solve such a complex problem, as it combines elements both from supply and demand, and more effort should be put in improving that proposal.

## Introduction

Health-care human resources (HHR) planning has been identified as the most critical constraint in achieving the well-being targets set forth in the United Nations’ Millennium Development Goals [1]. Moreover, the effective use and deployment of personnel is paramount to ensure an efficient service delivery in terms of cost, quality and quantity [2]. Failure to do so may result in an oversupply or shortage of clinical staff. While the former may lead to economic inefficiencies and misallocated resources under the guise of unemployment [3] or inflated costs through supplier-induced demand [4], the latter is linked to a more extensive list of negative effects, including but not limited to the following: lower quantity and quality of medical care as few resources exist to provide the necessary services and the visits are shorter [5]; work overload of the available physicians and nurses, resulting in sleep-deprivation, ultimately compromising patient safety [6]; and queues and waiting lists resulting from insufficient medical staff, causing avoidable patient deaths [7].

Another argument supporting HHR planning is the recent rise in health-care expenditure, both in *per capita* spending on health and as a proportion of *per capita* domestic product in real terms [8]. The average annual growth rate of health-care expenditure in a selection of 18 countries that are part of the Organisation for Economic Co-operation and Development (OECD) was 3.0 % between 1980 and 1990 and 3.3 % in the decade after [8]. Recent studies confirm the rising trend, with health spending growing at an average of 3.8 % in 2008 and 3.5 % in 2009 [9], well above the growth rate of the gross domestic product. Health worker wages account for about 50 % of total public and private health expenditure across several countries [5], meaning that cost containment and efficiency improvements will necessarily require the involvement of the workforce.

In sharp contrast to other scientific areas where a set of well-defined methodologies and techniques is generally adopted and refined to solve a given problem, in HHR planning, *methodologies* (the conceptual scope of analysis) and *approaches* (the techniques applied upon a particular method) abound, and there is still no commonly accepted or favoured procedure to accurately forecast physician requirements [3, 10]. The methodologies followed by countries vary significantly, in some cases with no long-term strategic HHR planning at all, but a wide array of options does not seem to be a determining factor in improving the accuracy of forecasting [11]. Despite the lack of focus, the accuracy of the projections appears to be making progress in some cases, as a review reporting the case of The Netherlands shows [12], an encouraging sign to the ongoing research.

A definite approach to the problem, or at least a stable starting block, will require a comprehensive overview of how the problem has been tackled since its inception. For this purpose, we provide a thorough analysis of the field, to lay down the foundations for future research, coupled with a historical perspective on the development of the HHR literature, analysing how the field has evolved and what methodologies have emerged and continue to be employed. Secondly, we analyse the strengths and pitfalls of each of the methodologies and provide a data requirement framework containing all the variables and data that need to be taken into account in order to address the problem thoroughly. The review is selective as it focuses primarily on articles that seem to have had a clear impact on the evolution of the field, although broad in scope as it attempts to extensively describe all known methods. Finally, it describes where we stand and the road ahead, providing a brief overview of new and emerging approaches to the HHR planning problem.

To the best of our knowledge, the last comprehensive academic paper on the subject dates back to 1978 [13]. Literature reviews exist but tend to either focus on a particular period or on a subset of the methodologies or techniques [11, 14] or to be framed as technical reports aimed at a wider readership, such as the OECD’s extensive review of 26 projection models used in 18 countries [9] or WHO’s policy recommendations to the EU [15]. The literature reviews can also consist of a technical report targeting a country in particular [16]. In fact, some authors point out that more systematic reviews, assessments of potential interventions and further research to aid policy makers are highly needed [17]. This paper aims to narrow this gap by being a starting point both for academics and policy makers.

### Literature search method

We carried out an extensive literature review, including academic research papers and technical reports from institutions such as the OECD or WHO. Selected papers date between 1951 and 2013, and the results were reported in a chronological and evolutionary way so as to clearly identify methodologies that are still in use to this day. The search methodology can be summarized as follows: after selecting a set of search terms and generating reliable combinations, we used electronic research databases to search for related articles. We then selected a maximum of 20 papers for each combination of search terms, including the 10 most cited, the 5 most recent and 5 that were randomly chosen. A backward/forward search was conducted, and the abstract was analysed to ensure that the papers met the search criteria. Papers that failed to meet any of the search criteria were excluded.

To identify search terms, we consulted the available literature reviews and technical reports [5, 10, 11, 13] so as to a obtain a list of key terms frequently used in this research field. Table 1 displays the search terms more frequently employed in the literature. Multiple combinations were selected using these key search terms. For instance, all possible combinations of *health* and *healthcare* with (AND) *workforce*, *manpower*, *physicians*, *nurses* and (AND) *forecast*, *projection*, *planning*. Related subordinate queries such as *physicians supply forecast*, *nurses supply forecast*, *healthcare supply forecast*, *healthcare demand forecast* were also employed. These terms were then used on the online databases PubMed, MEDLINE, Embase, ProQuest, Healthstar, ABI/Inform, INSPEC, Google Scholar and Scopus to obtain a base set of the 10 most cited, 5 most recent and 5 randomly chosen papers. Of this initial selection, an abstract matching and backward/forward search was conducted to assess whether the topic covered was relevant. Publications that failed to verify these criteria were excluded. A total of 308 publications were retrieved, with 75 meeting at least 1 of the inclusion criteria using the combination of search terms and were thus included in this review. Table 2 describes our search methodology.

**Table 1:** Key terms used to conduct the search

Keywords	Search queries
Health	Workforce planning
Healthcare	Healthcare forecasting
Workforce	Health human resources
Manpower	Health manpower
Physicians	Health planning
Nurses	Healthcare planning
Forecast	Health services
Projection	Health supply
Planning	Health demand
…	Healthcare needs
	Healthcare providers
	Physician forecasting
	Nurse forecasting
	Nursing staff
	Manpower
	Manpower planning
	Workforce forecasting
	Workforce projections
	Workforce management
	Staff levels
	Health staffing levels
	Shortage healthcare workers

**Table 2:** The search method applied in this review

Step	Search method
1	Identify common search terms from reviews, books and technical papers
2	Generate plausible combinations of terms to be used for search using the key search terms identified
4	Search for these terms on PubMed, MEDLINE, Embase, ProQuest, Healthstar, ABI/Inform, INSPEC, Google Scholar and Scopus
5	Select a base set for the results consisting of the 20 papers (10 most cited, 5 most recent and 5 randomly chosen)
6	Match the abstract and perform a forward and backward search to verify the relevance of the paper for the selected base set
7	Exclude papers that address none of the topics covered, that only make a brief reference to the subject at hand or that are not written in English

### Scope

HHR planning is a comprehensive field far extending the number of physicians and nurses. Other health-care workers such as hygienists, therapists, managers, administrative assistants and other support staff also play a critical role, relieving the clinical staff of bureaucratic and time-consuming tasks. In fact, skill-mix studies show that proper task delegation is critical to ensure proper health-care delivery. Furthermore, a complete assessment may also require the analysis of the impact of other indirect stakeholders, such as workforce educators, regulators, funders and employers. Assessing how the training is conducted (i.e. could the training time be reduced?; do medical schools have the capacity to train a given number of trainees?; are more medical schools necessary?), the impact of regulatory requirements (i.e. is the entry to medical school limited by government-fixed *numerus clausus*?) or financial and service constraints (i.e. can the existing hospitals and health-care units absorb a planned increase in the number of health-care professionals?) is a critical requirement for a well-guided policy.

Without disregarding the importance of these other professions, in this paper, we will focus solely on reviewing the planning of the clinical staff that directly provide health-care services and, more specifically, on the physicians and nurses, along with references to related fields like dentistry. Obtaining reliable projections for the available and necessary human resources is an obligatory starting point. Moreover, the prominence will be in the spectrum of different methodologies that may be used to obtain forecasts for the number of physicians and nurses, with short references to the approaches or technical apparatus, commonly used to apply a given methodology ^a^. Also, our concern is HHR planning only at the national and regional level. HHR planning at a local level (hospital or medical centre) is conceptually different, involving other methodologies and tools, and therefore, it is not inserted in this paper.

The remainder of this paper is organized as follows: in the “Background” section, we introduce the general and governing principles that characterize the health-care market. The background information provided is critical to equip the reader with the necessary concepts. In the “Evolution of the field” section, we proceed with an evolutionary and chronological description of the field, exposing the work and methodologies that have been shaping the research field. In the “Discussion” section, we discuss the current trends in this research area and the road ahead regarding future research directions. We also present a summary of all the findings, including a table with an overview of the methodologies and a data-requirement framework to understand which methodologies can be used based on the data available, as well as a proposal suggesting a way to develop an integrated approach. Finally, we finish with a brief summary and conclusion.

## Background

HHR planning as a scientific area and topic of theoretical and applied research evolved significantly from non-existence into a remarkable and serious effort of private and governmental institutions, which tried to anticipate how many human resources, primarily physicians and nurses, will be necessary in order to maintain or even improve the quantity, quality, availability and effectiveness of the medical services provided. Improved life expectancy and changing demographics, epidemiological trends, improved socio-economic conditions and an ever-increasing world population may result in a rise in the expected demand for health-care services [18] and, therefore, further additions to the list of patients of an ageing medical workforce [19]. It then comes as no surprise that health workers are recognized as a critical resource for achieving population health goals [1], working at the front gate of the health-care sector.

The health-care sector is an intricate, albeit fundamental, part of ancient and modern societies, and it comprises a long list of agents, from the individual seeking health-care services to the medical staff providing them, all operating within a legal framework involving providers, consumers, insurance companies, government, medical schools and regulatory institutions. Regardless of the statutory system in place, either a Bismarckian-based or a Beveridgean-based organization, at its core, the health-care market is always composed of both suppliers of health services and patients demanding their services. On the one side is the workforce of physicians, nurses and remaining clinical staff trained and ready to assist those in need. On the other side stand the forces that drive the demand for medical services, strongly related to demographic, socioeconomic and epidemiological factors. Analysing these two market forces is a critical step in assessing whether the available health-care human resources are enough in quantity and skills to meet the current and future demand in due time and may lay solid foundations for further research, considering perhaps changes to the existing health policy framework.

Despite the similarities, the health-care market diverges from a traditional market of goods and services for several reasons [20]. A high degree and extent of uncertainty affects both supply and demand; asymmetric information between physicians and patients, restrictions on competition, strong government interference and supply-induced demand are some of the most glaring differences that can be pinpointed. These may be relevant when assessing the impact of any policy involving HHR planning.

### Supply

Supplying human capital with the appropriate expertise so as to enable workers to perform and satisfy the demand for health care is no simple task. The time and effort required to equip HHR, especially physicians and advanced nurse practitioners, exceeds that of most other professions. In some particular health-care professions, the set of necessary skills to qualify for medical practice is acquired through extensive academic learning which involves the enrolment in long courses that may take up decades to complete due to a strict licencing process.

A considerable amount of HHR studies focus solely on this approach, basing their research on the estimation of the expected supply of physicians by accounting for the intakes, exits, migrations and population growth in order to maintain the present ratio of practitioners, using “stock-and-flow” models for that purpose [3]. The analysis of the medical *training* process is relevant but may be insufficient, as several other factors may affect the efficiency and effectiveness of the care services delivered.

Despite the limitations, some measures to overcome imbalances in the quantity (number) of physicians and nurses have already been identified in the health policy literature [17, 21], namely the following: increasing the number of domestic- and foreign-trained medical graduates or increasing the number of medical schools and classroom sizes; increasing the enrolment limits (*numerus clausus*); reducing the requirements for entry to medical schools; raising the wages of the medical staff, as well as the perspectives for their future career path; or reducing the costs of attending medical school, which may encourage potential students to enrol. In Table 3, we provide a more extensive list of policies to cope with a shortage in the number of health workers. These proposals are short-term measures to alleviate the immediate stress put on the health-care system triggered by an undersupply of personnel and may not be suitable for tackling long-term imbalances due to huge shortages or surpluses of medical staff.

**Table 3:** Health policy options for targeting health workforce imbalances and alter health-care outcomes (adapted from [17] and [86])

Field	Policy option
Education	Increase numbers of new students
	Recruit foreign graduates
	Recognize previous learning
	Improve curriculum content
Regulatory	Recognize overseas qualifications
	Introduce temporary employment regulations
	Subsidized education for return of service
	Enhanced scope of practice
	Different types of health workers
Financial incentives	Increase trainee salaries
	Raise wages
	Provide non-wage benefits
	Introduce incentives for return of skilled migrants
	Establish retirement policies
	Employ lay health workers
Professional and personal support	Better living conditions
	Safe and supportive working environment
	Career development programmes
	Public recognition measures

Still within the scope of supply, other approaches for handling the problem of insufficient human resources have also been suggested, addressing the problem from an angle besides medical training. For instance, the composition of the core competences and activities of the physicians, the *skill mix*, may be reorganized to enhance the roles performed by the clinical staff, relieving them from tasks that could be safely assigned to other health-care professionals [22]. This strategy does not require a change in the number of physicians but the restructuring of the available human resources and medical competences. Complementarily, supporting policies and reforms that enhance the *productivity*, that is, the ratio of output per unit of input given a certain level of technology and methodology, of the medical staff may result in an increased outcome that also does not require a change in the quantity of labour workforce [23]. Assessing the productivity of the clinical staff is now quite common [24], and operations research applied to the improvement of patient flows, queueing, master surgery scheduling, ambulance fleet management and staff rostering may play a very important role in increasing current levels of productivity. In summary, the initial focus of supply-based methodologies was on the training process. As of late, more focus has been given to the productivity and to the skill mix of the labour workforce as well.

#### Methodologies for modelling supply

Training (entries and losses) The purpose is to model the training process so as to predict the number of entrants in each year. This way, and in combination with migratory flows, mortality, exit and drop out rates, it becomes possible to estimate the number of physicians and nurses available for each year, with everything else held constant. Productivity The productivity of the medical workforce is not constant, as some professionals work harder or better than others or simply because there is an excess of bureaucracy to comply with. Without touching on the *quantity* of professionals, it is possible to reorganize services and incentives so as to promote increased productivity or implement lean and operations research recommendations to significantly improve the output and outcome of the workforce. Skill mix Since a degree of interdisciplinarity exists between medical professionals, it is possible to reassess the tasks performed by each professional, relieving physicians from day-to-day bureaucratic routines or reviewing the competences of the nursing profession so as to broaden their scope of action. Horizontal substitution (between different medical specialties) and vertical substitution (between different working classes) can be used to improve the amount of health-care services provided. Worker-to-population ratios This method establishes a desired ratio for the number of physicians and nurses per unit of population and compares it to the actual ratios. Policies to increase or decrease these ratios may then be pushed forward. Although simple and easy to apply as long as data is available, the method lacks the fine detail of such a complex system, ignoring other factors such as needs, demand or institutional frameworks that may have an influence on the productivity of countries or regions with similar worker-to-population ratios. Moreover, it abstains from exposing the causes for such asymmetries or from evaluating the efficiency of the available workforce.

### Demand

Demand for health care is a derived demand [25], which means that people do not seek health care services as a final good for consumption but as an intermediate service allowing them to be healthy and to improve their stock of *health capital* (well-being). They want to improve their health, and to do so, they seek health-care services. As in other markets, the determinants of aggregate demand for health-care services are population size, income and preferences. Moreover, for countries where medical care is mostly an out-of-pocket expenditure, demand is restricted by the patients’ ability to pay. If a patient requires medical attention and is unable to finance it, this *need* for health care will not translate into *effective* demand, despite its existence. Accounting for these cases is especially important in countries where health care is not publicly subsidized or where there are obstacles to entry other than the availability of resources.

**Fig. 1:**
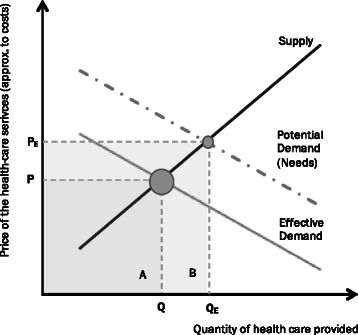
Law of supply and demand applied to health services. The health-care market depicted in terms of supply and demand, with a tentative distinction between *potential* and *effective* demand

The concept of *needs* in health care is not consensual in the health literature, with a semantic confusion arising from its use in health economics [13, 26]. While the economic or *effective demand* translates the actual, observed demand, usually measured in terms of service utilization ratios (such as bed occupancy rates, number of inpatients), the *needs* component tries to fully encompass the epidemiological conditions that characterize a given population, measured through morbidity and mortality rates or by the opinion of a panel of experts, and how that may translate into a given quantity of required health-care services. Therefore, we see that the classical concept of economic demand may not reflect the biological needs of the population, as it may leave out the necessities of the population regardless of their ability to pay. In the needs component, the emphasis is on the medical conditions that may lead to demand for health care, deriving from the evolution of chronic diseases, prevalence rates and overall morbidity patterns. This distinction is better illustrated in Fig. 1, where we present the case when all demand is met, at a given price, and equilibrium is attained. Theoretical demand, projected strictly in terms of biological needs without a budget constraint (either households’ income or public budget), may not always correspond to the demand effectively observed. The reason being that the quantity sought is limited by the disposable income directed towards out-of-pocket health expenditure or by limits to the government budget that is allocated to health care. We draw the distinction by plotting both the curve of *needs* (potential demand), corresponding to a *no gap* scenario, and the *economic* (*effective*) *demand* that is actually observed.

Although needs is a fundamental concept, it should not be decoupled from economic demand, as it should not ignore the budget constraints of the economy. In fact, the country may not have the ability to provide all the health-care services presumed to fully satisfy needs. If the area delimited by B (cf. Fig. 1) is larger than the domestic product of the economy, it will be impossible to meet all the perceived health-care needs of the population. Like any other problem involving scarce resources, a serious analysis should not abstain from recognizing the existence of financial impediments. Conversely, it should try to quantify needs, serving as a theoretical benchmark for the future.

This has not always been the case. Some studies estimate demand solely based on the current level of service in relation to future projections of demographic profiles [27, 28], thereby leaving out an important determinant of demand, the epidemiological needs [29, 30]. When and how disease trends evolve is critical to properly anticipate the needs of the population, a proxy to the expected future demand. For instance, chronic diseases have been increasing globally [31]. China, a country usually not associated with overweight and obesity problems, has experienced an upsurge in type two diabetes. According to the data reported, in 1980, less than 1 % of Chinese adults had diabetes, but by 2008, the prevalence of the disease had already reached 10 % of the population [32]. As a result, it is expected that more endocrinologists will be necessary to assist with the treatments. The raw definition of needs is not subject to any boundaries other than those set by epidemiological constraints and medical advances.

A substantial part of the studies targeting supply hold current demand constant, thereby leaving out a proper analysis of what drives demand for health care. In fact, a change in the factors that influence demand or the emergence of new health conditions in a population may require a reorganization in the quantity, composition and skill mix of the medical workforce to ensure that all supply meets demand. This suggests that targeting the right number of people and the right skills depends as much on the health conditions and epidemiological characteristics of a given population as on the supply of physicians and nurses [33].

In summary, three methods are commonly used to analyse HHR planning from a demand-based perspective [13]. Most of the methods build upon the definitions of needs and effective demand, and some overlap in their scope of application. Contrarily to the approaches found in supply-based methodologies, where the object of study remains the same and alternative analytical methods are employed, in demand, opting for a different method may change the scope of the analysis.

#### Methodologies for modelling demand

Needs (or potential demand) This method determines the effect of health diseases, epidemiological patterns and overall mortality and morbidity rates in the demand for health services and obtains an approximate number of personnel hours required to cover those needs. Needs are usually assessed by a panel of experts in epidemiology and may not match the services that the public wants. Economic (or effective demand) In this method, we look at the services actually contracted by the population, subject to the usual economic constraints that may put an upper bound on the quantity solicited. In sharp contrast to the first method, effective demand may not imply a healthy population, especially for poor countries without a subsidized health-care service since the general citizen lacks the means to obtain health-care services. The method ignores needs or wants and assumes that all the remaining variables remain constant, although that requirement may be relaxed by complementing the results with other methods. Service targets Service targets extend a needs-based approach by incorporating other measures, such as consumer needs, in order to establish service-target ratios to be accomplished. Service-target approaches decouple the multiple areas of health-care services and proceed with an independent analysis of each subsystem, with the main advantage being a more detailed proposition of the changes required, with separate recommendations for distinct areas.

## Evolution of the field

Although the health workforce has long been a concern to policy makers, including those of ancient Rome [34], the first academic research articles discussing manpower planning in general, and health-care workforce planning in particular, date back to the 1950s. This was a natural response to both the creation of national health-care systems and universal insurance schemes.

A universal health-care system with no exclusion based on preconditions and with no restrictions on access, an idea put forward by Bismarck in the compulsory social insurance form, and promoted by Beveridge as a national health service [35], requires a well-prepared and readily available team of physicians, nurses and administrative staff. To ensure that services are in fact provided, public medical universities were created along with subsidized access to medical training. These reforms resulted in the emergence of a national ecosystem of health-care suppliers and a pool of patients, a significant change from the decentralized network of health-care providers. The ubiquity of access required providers to be distributed evenly so as to satisfy the needs of the population.

After this period of sustained and prolific economic growth, a period of crisis followed. Expectably, the economic slowdown put the focus on efficiency, towards a better use of the available resources. During this period, many developed and developing countries experienced shortages of health-care providers, mostly nurses [36], justifying the growing interest in this newborn academic research field.

This was the period when the first articles on health-care workforce planning emerged. We separate the analysis of the unfolding of HHR planning into three separate stages, corresponding to the evolution of how the health-care worker is perceived as an object of study [37]: (a) the health worker as a production factor, (b) the health worker as an economic factor and (c) the health worker as a necessary resource. This structure is helpful in the sense that it exposes the role given to the workforce, once studied as an inorganic fixed-input factor and more presently viewed as a complex and necessary resource with its own idiosyncrasies like any other economic agent.

### First phase: factor of production

The first articles published on the subject date back to 1950, with HHR planning being perceived as a production function, where the labour workforce is an input factor. The research, triggered by general health worker shortages in developed countries [38, 39], led a growing and diversified body of research that diverged into different approaches. Not surprisingly, some of these articles are the result of initiatives promoted by governments and international organizations to address their own domestic shortages of physicians and nurses, while others are *ad hoc* contributions of attentive researchers keen on providing an insightful contribution. The techniques employed vary from descriptive to predictive or merely comparative techniques and usually involve econometric regressions, static tables, linear programming or benchmarking. These techniques are then applied to the areas of analysis previously described, either supply, economic demand, needs and service-target or worker-to-population ratios, which we will identify next.

A significant part of the research papers produced at that time are well-documented, with comprehensive lists and reviews of the models developed still available [40, 41]. Of these, we highlight those that are still cited in the literature and available online.

#### Supply-based methodologies

The very initial concern of those conducting HHR planning was estimating the necessary number (head count) of medical professionals to either maintain the current worker-to-population ratios or reduce/increase it if an imbalance was found. One of the first insights into the evolution of the supply of physicians was done by crossing the observed physician-to-population ratios along with the posited population growth in the United States of America, by that time impulsed by the “baby boom” and by an expected increase in the use of medical services. The people in charge of HHR planning evaluate the number of physicians required to maintain the ratios given those demographic and economic changes [42, 43]. In the report, the same criterion is used to estimate future manpower requirements for all the available medical specialties, nurses and miscellaneous professions necessary for due operation.

One way of doing so is to look at the current stock of professionals and factoring in negative and positive flows that affect the stock. Factors such as mortality, migration or retirement generate losses to the current workforce stock. Likewise, entries from medical schools and immigration increase the current level of professionals. Models that map this structure are commonly known as “*stock-and-flow*”. Despite not using this specific terminology, models created at the time already incorporated the idea of increases and decreases in the current stock due to exogenous factors and then used that information to obtain projections [44–46].

Focusing particularly on the supply of nurses in the United States of America, other papers proceed with an analysis of the economic factors, namely the hourly wage and the wage of the nurse’s spouse and the effect on the supply of nursing professionals [45, 47]. Evidence suggested that hospitals exercise monopsony power, which has an impact on how a supply gap may be tackled. Moreover, results also suggest that the cost of paying wage incentives to increase working hours is considerably smaller than the cost of training additional professionals, something to take into consideration when evaluating HHR reforms.

The product of this novel research was tested in the field. For instance, in the analysis of the health-care workforce in Taiwan, estimates for the supply were generated on the basis of retirement, migration and death rates applied to graduations. They incorporate the training process and its effect on the supply of physicians [48].

*Methodologies*: Training (entries and losses) [42–46, 48], Productivity [45, 47], and Worker-to-population ratios [42, 43].

#### Demand-based methodologies

One of the first publications in the field of HHR planning starts by differentiating the aforementioned dimensions of workforce planning [49]. Klarman et al. argue that, although medical *needs* could form the basis for determining workforce requirements, it cannot be decoupled from economic costs, an active constraint to the extent, scope and applicability of reformist policies. A forecast of the necessary supply of physicians is not provided, but it is suggested that the shortages in the specialty areas may be a sign of an overall supply shortage.

Another way of predicting the necessary future hospital beds is by extrapolating from a set of factors assumed to drive the demand for health care, namely socio-economic factors and biologic needs, measured through morbidity rates [50]. This approach was also used to estimate hospital bed requirements, providing both empirical works on real data for the United States [51] and theoretical frameworks with hypothetical parameters [52]. In some cases, the approach of forecasting bed requirements would be extended to other health-care units such as primary medical care, nursing home care, consultant medical care (medical care provided by a physician with specialized training), hospital care or domiciliary care [52].

Methods for estimating the number of professionals required (head counts) from a demand perspective also started emerging at around this time. For instance, in one case, estimating the number of necessary physicians for the future was done by calculating the number of professionals necessary to close the gap between observed and unattended demand, where demand is measured in terms of utilization. In this case, using service-level indicators again for the United States [53].

In other studies targeting the U.S.’s health system, the influence of exogenous variables such as age, income and urbanization is used to extrapolate the effect of dependent variables on health policy and HHR planning, including the number of persons with health insurance, the number of general practitioners, medical specialists, available short-term general hospital beds, admissions and mean duration of stay per case [54]. This approach is also similar to the one used in two other models, the first using data aggregates to facilitate HHR planning at national, state and substate levels and the second going to the level of detail of the individual and his interactions with professionals and institutions [46].

More comprehensive approaches to estimate economic (effective) demand were also addressed. Some papers suggested incorporating indicators such as an increase in population, economic development, improved education, a change of supply, age distribution and other unpredictable factors. Simple calculations, such as the ones used in the former Soviet Union, could be performed by extrapolating based on observed norms of practice regarding the number of patients attended and then complemented with basic biological needs by incorporating data about morbidity and mortality rates [44]. Methods like this were then applied to countries such as Taiwan, characterizing current public and private sector demands for health services [48].

Another option for measuring demand also elaborated during this time consisted of using other indirect indicators, namely short-stay services, services of nervous and mental hospitals, physicians’ services outside hospitals, dental services and other health services. The data is then fed into a model that tries to minimize the gap between the number of individuals employed in medical services that attend to the demand for personnel in that occupation [53]. Estimates were generated for the United States.

Finally, it should be noted that attention was constantly being drawn to the importance of prevailing morbidity, a basic indicator for assessing medical manpower based on a needs-based approach. Some authors stress that it is the hospitals and their internal need for residencies that actually determine the number of specialties [55]. This may not reflect with accuracy the actual needs of the population since patients could potentially remain unattended or in long waiting lists, but it is an insightful indicator if waiting lists are also factored in. Finally, they also consider the specialty of the physicians’ role, warning that general practitioners fulfil key medical functions and should not be relegated to second place. The concept of *skill mix*, despite not formally and explicitly defined, is here put in evidence.

*Methodologies*: Needs (potential demand) [44, 46, 48–50, 55], Economic (effective demand) [44, 46, 49–54], and Service targets [46, 53].

### Second phase: economic agent

The first phase of HHR planning was characterized mainly by an aggregate analysis of the health-care market, with independent and/or cross-analysis of supply and demand. Reviews produced at that time refer essentially to needs- and demand-based approaches, as well as simple worker-to-population ratio benchmarks [56]. The phase that starts in the late 1970s and goes onward through the 1980s and 1990s redefines the role of the HHR, previously seen as an homogeneous input factor, into a complex economic agent [37]. The adoption of such perspective broadens the scope of analysis, namely by assuming that health-care workers react to economic incentives.

The deepening of the analysis is done through the application of microeconomic theory to the study of health labour workforce, thereby exposing dimensions that had gone unnoticed when looking only at the aggregates, although a macroeconomic analysis continued to take place [57]. It was triggered by two macroeconomic observations occurring at this time [37]: a perceived oversupply of physicians and nurses [58–60] and an upsurge in health-care expenditures [8]. During this phase, attention was given to topics such as health worker licensure [37, 61], information asymmetry distortions [62] and its potential repercussion as an unnecessary increment in demand induced by health suppliers [63] and health worker performance and productivity [64]. Furthermore, HHR planning became a major concern in related fields, such as dentistry [65].

#### Supply-based methodologies

Although the previously mentioned topics are of notable relevance, some have no direct utility in the elaboration of projections and forecasts of future health-care needs, serving only for policy guidance. For that reason, we will concentrate our efforts on the performance and productivity of health workers, a method fully within the umbrella of supply. In terms of policy, it is less demanding to put in practice as it does not require structural changes to the training process or to medical schools. In theory, more people can be served with the exact same amount of human resources if only their productivity increases. Improving the efficiency of the available pool of resources is therefore an attractive methodology.

This is the line of research followed in a paper where a microanalysis of the factors that may influence the output (and therefore productivity) of the health workers is conducted, in particular nurses in the United States [47]. Sloan et al. found that there is a strong supply response to the hourly wage. Raising the hourly wage is, in fact, their proposal to respond to a short-run supply shortage, arguably a quicker response than changing the number of intakes to nursing schools. Taking another route to reach the same goal, one study tries to undercover job satisfaction indicators and perceived productivity in 24 hospitals for a staff nurse population [66]. The purpose is to understand the factors that may raise productivity but also to find a connection between job satisfaction and the quality of care provided. Similarly, waiting and distance times can also be used to assess the physicians’ productivity, a study conducted using data from the United States [67].

In the same line of research, some authors conducted an observational study of 56 physicians in order to uncover the factors that may influence productivity, measured as the ratio between the number of patients seen per physician and the time spent with the patient [24]. The main research question was understanding which factor contributed the most to the variance in productivity: the patient or the physician. Results suggest, according to the study conducted in a Veteran Affairs’ medical centre in the United States, that the individual physician explains the variations in productivity observed, with the actual patient playing a minor role. Similarly, in another study also conducted in the United States, the productivity of physician assistants and nurse practitioners and their role in the health-care workforce is analysed [68]. Scheffler et al. find that these two categories of health workers could have a significant influence on the future health-care workforce if some vertical and horizontal substitution occurs and tasks are delegated. Note that the change of setup hereby suggested tackles productivity from a different angle: instead of raising the output, the inputs are altered.

*Methodologies*: Productivity [14, 24, 47, 64, 66–68] and Skill mix [68].

#### Demand-based methodologies

Studies focusing solely on the demand side produced during this phase are considerably less common than in the first phase. The ones that do so are more concerned with the lack of attention given to the importance of biological needs. It is interesting to note that, at the turn of the decade and in subsequent years, a lot of emphasis is again put on the *needs* of the population. Some authors suggest a needs-based evaluation as a requirement to produce accurate forecasts [29, 56]. This option contrasts with that of other authors, which propose using benchmark as a viable alternative to potential or effective demand projections [69]. The work developed consisted of comparing the number of active physicians *per capita* in the United States, adjusted for population differences between similar locations, without uncovering the causes for the given asymmetries.

Assessing the needs of the population was also the method of choice in the dentistry field to calculate oral health workforce requirements. In particular, needs were projected by the amount of oral care, including preventive, special group care, surgical, orthodontic, periodontal, restorative and prosthetic, that different age cohorts would require [70]. Then, the time necessary to treat each of these conditions is estimated, and the number of dentists to perform those tasks is derived. Also applied to dentistry but with a focus on the skill-mix distribution, productivity changes are estimated by examining role substitution in dentistry [71], helping to conduct evidence-based scenario analyses in The Netherlands.

*Methodologies*: Needs [29, 70], Skill mix [71] and Worker-to-population benchmarking [69].

#### Integrated methodologies

A new strand of the literature also emerged during this phase covering supply while at the same time considering projected changes to demand. In a review of supply projections conducted both in Canada and in the United States [14], the authors argue that the traditional supply projection methodology that characterizes the licensure cycle and productivity metrics is incomplete if unmet *needs* of the population are not defined and included as a clear research goal, as well as economic, financial or infrastructure resource constraints.

The integrated approach is also present, for instance, in the implementation of the “System for Health Area Resource Planning” (SHARP) [72]. This analytical framework combines all the major methodologies: it includes the socio-economic factors that drive economic demand, morbidity and the remaining epidemiological factors that drive needs, the formation process of the health-care supply of workforce and utilization rates in order to incorporate the current use of health-care services. The framework was successfully used to support HHR planning in Canada, especially in the province of Ontario, reinforcing the idea that an integrated or systems approach, combining the multiple facets of the problem, is the way to go in the future.

*Methodologies*: Integrated [14, 72].

### Third phase: fundamental resource

In this phase, the notion of health labour workforce is reformulated, this time viewing it as a necessary resource. From the 1990s onto the 2000s, the emphasis is on the regional asymmetries in the placement of the workforce and in the migration flows from developing to developed countries [37]. All models proposed include both supply- and demand-based methodologies to tackle the problem.

#### Integrated methodologies

Methodology-wise, the trend observed is a continuation of the second phase, with the call for a holistic approach to the problem. HHR planning must be addressed from an integrated perspective, including when analysing all the blocks of the functioning system so as to calculate the current and future *gap* between supply and demand [73]. The authors’ proposal is in line with the SHARP framework: modelling key demand (economic and epidemiological) and supply inputs. Furthermore, it is continuously stressed that the epidemiological drivers of the need for health-care services should always be part of HHR planning [30, 74].

When looking at the research literature produced at the turn of the century, this trend becomes clear. Summing up the results achieved so far, we can see that health-care workforce planning is a complex endeavour, and it becomes necessary to identify all the relevant variables to accurately forecast the necessary resources for the future [75]. Again, these variables relate to supply and needs methodologies. A practical work conducted in Lithuania to forecast family physicians for a 10-year timespan employs this approach [76]. Firstly, this approach calculates the supply of physicians through the usual process of modelling the training of physicians. Moreover, it crosses the supply forecasts with three different projections for demand: firstly, the requirements established by a panel of experts using a Delphi technique; secondly, the resources necessary to increase the number of visits; and thirdly, an upper bound placed on the worker-to-population ratio so that one family physician serves no more than 3 000 inhabitants. The conclusions reached suggest that the well-informed panel of experts elaborated the most accurate projection of demand for family practitioners and that none of the supply projections was right on target. Similarly, in a forecast analogous to the nursing profession in Germany, the analysis is extended from the usual supply and demand to include the effects of occupational flexibility and employment structure. Adding these two elements to the analysis has a relevant influence on the projections [77]. Notably, this pensiveness with the organizational role, where the HHR is more than an aggregate number but rather a dynamic and complex sum of individuals, is clearly gaining traction.

In the same line, some researchers suggest a needs-based analytical framework that incorporates input from four separate elements: demography, epidemiology, standards of care and provider productivity [30], again falling in the realm of integrated approaches. Alternatively, needs can be decoupled in a functional form so that service targets can be defined and deployed [1]. Dreesch et al. claim that methods focusing strictly on the supply, on the demand or on both fail to address or recognize the effects of the skill mix (the potential of substitution) between health professions. The importance of a more integrated approach to HHR planning is also restated. With more or less variables, the trend is clear: recent models use information from both demand- and supply-based methodologies, including inputs as varied as demography, the training process, workers’ productivity or biological needs in order to generate their forecasts [18, 78, 79].

Although the emphasis is fundamentally put on addressing the problem from an integrated perspective, new strands of literature were also developed during this phase. For instance, it is suggested that instead of addressing the problem from a quantitative perspective, either by adding to or subtracting from the stock of health workers, it should rather be addressed with internal reorganizations, redefining which tasks can be performed by whom [80]. Such internal substitution and activity delegation could be executed by transferring skills from the medical specialist and the general medical practitioner to other health professional roles, namely nurses with higher education (midwives) or by creating new roles. This methodology involves, therefore, playing with the *skill mix* of the health-care professionals. This was put in practice in Ireland by employing a model that targets both supply and demand, reflecting the concerns for including all parts of the system [28, 81]. Moreover, it tests four policy interventions, three of which related to supply and the last related to the skill mix: increasing vocational training places, recruiting professionals from abroad, incentivizing later retirement and increasing nurse substitution so that nurses can deliver more services. Similar studies, encompassing the workforce supply, demand and the skill mix, were also conducted in the dentistry field during this phase [82]. In this case, workforce supply and demand for oral health needs are projected to study the impact of skill-mix reorganizations. To forecast future dentist numbers, a simple percentage increase based on previous yearly increases is considered. To estimate demand, demography evolution, rates of edentulousness, patterns of dental attendance and treatment rates of older people, as well as general dental service treatment times, are considered. The effect of the skill mix is then studied considering several scenarios of varying skill-mix use. Gallagher et al. find that widening the skill mix can be extremely helpful to build capacity for dental care.

Another concern that is raised during this phase is that of measuring the *outcome* as an important indicator for assessing the quality of the health-care services. The outcome is a fundamental indicator for HHR planning. In particular, equitable and timely access to health care are a precondition to a good outcome, which is the variable to be maximized [83].

In summary, it can be said that this stage was a phase of settling with methodologies, namely supply-, demand- and needs-based approaches, and of urging for a more integrated approach while paying attention to the roles of each health professional and the degree of substitution between professions. Furthermore, a concern about the *outcome* of health-care services was raised, where effectiveness and quality of the treatment is considered on par with the number of patients seen (productivity).

*Methodologies*: Integrated [18, 18, 28, 30, 73–79, 81, 82], Skill mix [1, 28, 77–82], Needs [30, 77], Service targets [1] and Productivity [77–79]

## Discussion

**Fig. 2:**
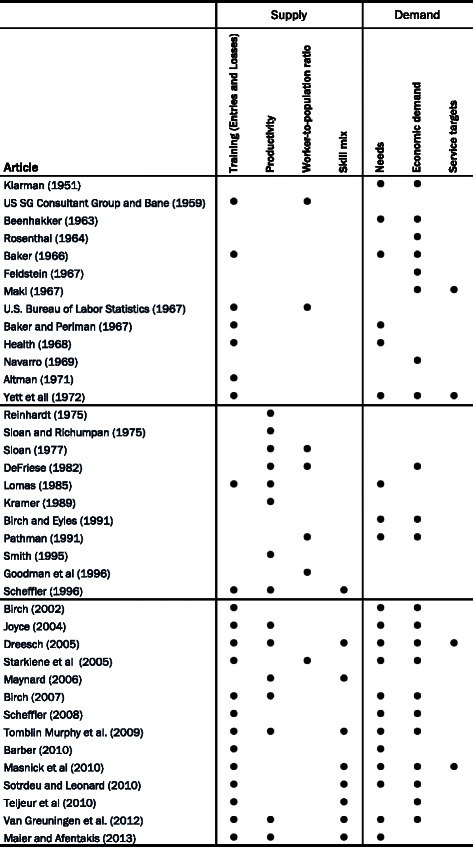
Identification of the conceptual methodologies found in some of the literature for the period of 1950–2013

Five decades of work in HHR planning fuelled by eminent global shortages of health professionals have contributed to establishing this research field as an important scientific area, decisive for achieving worldwide health-care targets [1]. Significant results have been attained. In particular, new methods and techniques were developed, and the accuracy of projections improved remarkably [23], and HHR planning became an area of prominent interest, with the number of publications in the field increasing over the years. Moreover, the literature evolved, replacing some approaches with others, paying more attention to the health-care workers and their productivity and to the delegation and distribution of skills. It prioritized integrated approaches and the role of epidemiology in addressing the problem. In fact, when we look through all the methodologies reviewed (Fig. 2), the emerging trend clearly supports this claim. Integrated approaches are gaining ground after decades of partial analyses turning to either a supply- or a demand-based approach and in its simplest form only resorting to worker-to-population ratio benchmarks.

In Table 4, we summarize the methodologies and describe the necessary assumptions for using each of the approaches, along with their advantages, limitations, how these limitations are overcome, requirements and the countries in which their usage was documented (according to [9]). In the past, this overview would probably help in choosing the methodology to adopt. With the call for more integration, it assists in showing how a methodology may fill in the gap towards a cohesive framework. Also, it serves to show that there is no perfect methodology capable of providing accurate forecasts without considerable pitfalls and that there is a trade-off between simplicity and completeness, where going for a simpler methodology may implicate leaving out important parts of the problem.

**Table 4:** The methodological approaches established during the first phase of research

Methodology	Description	Assumptions	Advantages	Limitations	Overcoming limitations	Requirements	Documented usage^a^
Supply							
Training	Projects the availability of health-care professionals based on the current stock of clinicians, the training process (entries and dropouts), migration flows, attritions and retirement rates	Demand for medical services is assumed to remain constant and the projections are used to reduce the supply gap	Predictions for the future supply can be obtained in a fairly simple and immediate way	Demand for medical services is assumed to remain constant, which may not be true No critical assessment of the adequacy of current service levels	Incorporate a model of demand: economic or needs-based (or both)Evaluate current level of service through waiting lists, overtime hours,foreign workers, etc.	Accurate and up-to-date accounting of the current stock of physicians and nurses, migration rates, entry and drop out rates and expected retireesService usage levels from the health-care sector	Australia, Belgium,Canada, Chile,Denmark, Finland,France, Germany,Ireland, Israel, Japan,South Korea,Norway, Switzerla nd,The Netherlands,United Kingdom,USA
Productivity	Reorganize services and/or economic incentives to promote higher productivity. Work harder or work smarter	Physicians and nurses act as rational agentsan d react to economic incentives like wage increases	Does not require a change in the quantity of human resources. Can be implementedimmediately	Productivity improvements may not be enough to accommodate large gaps in the supply of professionals	Do not preclude from evaluating the number of professiona ls necessary given differentproductivity levels	Operational indicators like the number of patients served with a given number of FTEs (or head counts)	Australia, Canada,Japan, Korea, Netherlands, N orway,Switzerland, UnitedKingdom, USA
Skill mix	Delegate certain tasks to other health professionals. Substitution can be horizontal (betweenmedical professions) orvertical (betweenphysicians and nurses)	Professionals can assume new roles and perform new tasks	Does not require a change in the quantity of human resources. Can be implemented immediately	Enforcing such changes can be a political challange.Does not solve large gaps in the supply	Providing success stories to involved stakeholders, healthauthorities and medical associations	Education schools that can provide advanc ed education to the existing workforce	Netherlands, United Kingdom
Worker-to-population ratios	Specifies desirable worker-to-population ratios based on direct comparison with another region of country	Regions and/or countries can be directly compared	Extremely easy to understand and apply Useful forproviding baseline comparisons	Does not take into account the intrinsic differences between regions a nd countries, the productivity and s kill mix of the avail able workforce	Does not take into account the intrinsic differences between regions and countries, the productivity and skill mix of the available workforce	Records of the current workforce to population ratios	Chile, France, Ireland, Israel, Switzerland,United Kingdom
Demand							
Economic	Estimates future requirements by projecting the effect of demographic and socio-economic factors on the current level of service	Current level of service is adequate.Skill mix and distribution of health service is appropriateDemographic profile of the population and its effect on health-care demand can be accurately forecasted	Conceptually easy to understand and to apply Allows decoupling of the various components of demand and their influence on the overall aggregate demand	Tends to produce estimates of HHR demand that exceed practical limits No criticalassessment of the adequacy of current service levels Ignores the real demand,focusing instead onthe effective demand	Take financial constraints into considerationEvaluate current level of service through waiting lists, overtime hours, foreign workers, etc.Include a needs-basedevaluation	Accurate and long-term demographic estimates Service-usage levels from the health-care sector Macroeconomic indicators and stati stical data crossing income and usage	Australia, Belgium, Canada, Denmark, Finland, Germany, Japan, Norway, South Korea, Switzerland, The Netherlands, USA
Needs	Considers the effect of epidemiology on the demand for health-care services Projects age- and gender-specific needs based on morbidity epidemiological trends	All health-care needs can and should be metResources are used in accordance to needs	Allows for a fine-gra inedanalysis of the requirements of each medical specialty Is independent of the current service-utilization ratios Easy to understand	Absence of ec onomic/efficiency considerations mayren der the projections unattainable Dependent on epidemiological projections which may not be obviousDoes not consider the current level of provision nor the capacity of the country to deliver health care	Consider an upper bound for a practical resultConsider projections of the most common he alth patterns Incorporate econo mic considerations in the model	Demographic esti mates that are accurate Service-usage levels from the health-care sector	Belgium, Canada, Germany, United Kingdom
Service targets	Defines normative targets for the production of health-care services, which are then converted to HHR requirements	Assumes that established service targets are achievable in terms of financial and physical capital resources	Easy to d efine, interpret and understandFacilitates cost estimation Requires modest data and planning capabilities	May originate unrea listic assumptionsIgnores financial and other active constraints	Incorporate economic considerations in the model	Current level of service	

^a^OECD Report

Source: adapted from Hall and Mejia [13], O’Brien-Pallas [11] and Dreesch [1]

### An integrated approach

The importance of a comprehensive, integrated approach is continuously emphasized throughout the period in review [3]. Although the need for an integrated approach had already been stressed in several past publications, it keeps on reappearing, suggesting that it might not have been fully addressed as of yet. This approach faces many challenges. A dynamic, system-level perspective covering key drivers of supply and demand that includes both manpower planning and workforce development is critical to overcome such challenges [81]. The importance of paying attention to needs is also continuously stressed, as changes in the health patterns of the populations take place [84]. In summary, integrated approach refers to a method that incorporates in its process projections of the workforce supply and the impact of microeconomic and organizational changes in productivity and in the skill mix, of the evolution of demand for health-care services and also of the evolution of health diseases and its potential impact on the health system.

**Fig. 3:**
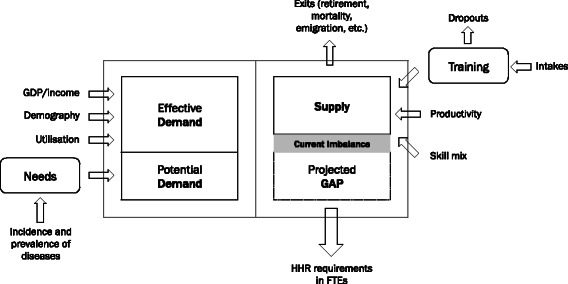
An integrated system that incorporates several methodologies to address the many facets of HHR planning

Notwithstanding, integrating all the pieces may be a puzzling task. To assist with the task, in Fig. 3, we provide a high-level functional diagram with a proposal for how methodologies could be coupled so as to turn it into a seamlessly integrated system. On the supply side, we have the current stock of workers along with the training process so as to obtain an initial snapshot of the current workforce. The current stock, which may or may not be enough to tackle current demand, in which case an imbalance exists, is subject to positive and negative flows that may alter its number and composition. This given quantity of workers may provide more or less health-care services depending on their productivity and skill mix, and that influences the conversion from head counts to full-time equivalents (FTEs). Such conversion is critical to properly assess the health-care workforce, as a significant number of physicians and nurses work part-time only. For this reason, FTE is a more accurate measure as it normalizes the head counts. On the demand side, economic (effective) demand can be initially measured by analysing utilization indicators. How this demand will evolve in the future will then be subject to typical economic factors such as demography and the growth of the income/GDP. In parallel, potential needs can be assessed by incorporating incidence and prevalence of diseases and then mapping a given disease to an estimate of FTE requirements. Whether future supply forecasts should tackle all of the estimated needs is a decision left to the consideration of the policy maker, as this analysis does not incorporate financial constraints. Such an integrated approach is more complex, but not necessarily more difficult [12]. In fact, policy-making cannot abstain from factoring in financial and service planning considerations in a *post hoc* analysis, since there may not be enough resources to accommodate for a sudden increase in the number of professionals. Such analysis is not limited to a money perspective, to the financial burden inputted on the system for educating and hiring these medical professionals or to the installed capacity in terms of medical schools, university hospitals, hospital beds, primary care facilities and others, in order to absorb planned increases in the health-care services labour market.

### Data requirements

None of these methodologies can be applied without the adequate data to feed the model. A bare minimum of information regarding the available medical workforce is always required. Table 5 summarizes the most important indicators for conducting a proper forecast. It is not strictly necessary to possess all the information listed, but the availability of the data increases the probability of a more comprehensive projection.

**Table 5:** Data requirements for making use of each of the different documented methodologies

Methodology	Indicators	Data requirements
Supply	Stock of licensed providers Baseline stock, age/sex distribution, growth projections	High
	Annual additions to licensed stocksGraduates, in-migration (foreign-trained, immigrantes, on temporary work permits), returned to profession	
	Education/training programmesNumber of programmes and students enrolled, attrition rates, years to complete programme, number of graduates, costs	
	Annual attritions to licensed stocksRetirements, mortality, career changes, emigration, abroad	
Productivity	Labour market Occupational participation rates, occupational employment rates, employment projections, vacancy rates, turnover rates, wage rates, productivity growth, cyclical factors, alternative career options	High
	Employment statusFull-time, part-time, casual, full-time equivalent (FTE), average hours worked, direct patient care hours, no longer practising, not licensed in jurisdiction	
Skill mix	Government policy variablesHHR education funding, alternative delivery modes, licencing regulations, professional roles/deployment, recruitment/retention strategies, immigration policy, remuneration rates/types, HHR capacity-building	High
Worker-to-population ratios	Health labour workforce Number of active and employed physicians and nurses	Low
Economic	Population demographicsTotal population, age/sex distribution, births/deaths, population projections	High
	Socio-economic variablesDisposable income, GDP growth projections, ethnic factors	
Needs	Population health statusAge/sex mortality, morbidity, acuity	High
	EpidemiologyIncidence and prevalence rates, hospital discharges, health patterns of the population	
Service targets	Utilization patternsNumber of occupied beds, number of inpatients and outpatients, number of surgeries/screenings/consultations performed, etc.	Low to high

Simpler approaches require fewer data. Worker-to-population ratio benchmarks require a head count of the number of licensed medical professionals, usually made available by the government, medical and nurse associations or by unions. Service targets use the current level of service, which can be obtained from the hospitals’ operational key performance indicators. Needs (potential) and economic (effective) demand, on the other hand, require a more extensive set of indicators. For needs, it is necessary to assess and validate current and future incidence and prevalence of diseases and how that may convert into necessary resources. Both tasks are not straightforward and usually require acclaimed experts in epidemiology to step in and provide both the estimates, as well as an accounting of the resources that will be necessary. Effective demand makes it necessary not only to obtain metrics similar to those indispensable for a service-target analysis (such as the number of inpatients and outpatients, number of occupied hospital beds, average length of stay) but also demography and socio-economic projections and how they affect demand. Finally, modelling supply is also a challenging task in terms of data requirements. Unless evidence is found showing that the worker-to-population ratios will remain constant for a long period of time, a supply-based analysis must be factored in. In such a case, it is necessary to know the current stock of licensed providers, as well as the number of intakes, exits and annual attritions, which makes it necessary to model the training of medical professionals.

Assuming that developing countries are in possession of fewer data and that developed countries have more information available, methodologies that require an extensive set of data will be difficult to implement in developing countries. Therefore, such countries may start by using simple techniques such as the worker-to-population ratio or service-based benchmarks to tackle their present imbalances. Developed countries should continue collecting data and enhancing their models, adding less tangible and yet relevant dimensions, such as productivity or skill mix if they are not present already.

## Conclusion

In this paper, we reviewed over 60 years of publications in HHR planning. While doing so, we observed the evolution of the field, when and how methodologies emerged, how they have been applied and the robustness of the results, and we also identified the current trends in the field. This work was called for because there is still no accepted methodology to address HHR planning. Given the rampant costs in the health-care sector and the overall influence that health care has on the general welfare of society, as well as the potential impact of shortages on the worldwide supply of medical professionals, an assessment of what has been done and achieved and what remains to be done was necessary to properly guide further developments in this relevant field. Moreover, when we contemplate the complex training process required to earn a licence as a practitioner, we understand that a shortage in medical professionals cannot be accommodated fast enough by decree, either by increasing the number of intakes to medical schools or by inviting more foreign-trained doctors or nurses.

Despite the abundance in approaches and techniques to determine supply and need for professionals, none of the methodologies has ultimately proved to be superior [85]. Recent studies testing current forecasting models show that there is still plenty of room for improvement given the gap between projected and actual results [12].

It becomes even clearer that workforce planning should be accurate and performed in due time given the attritions and the delays in enacting policies in the health-care sector. Adapting medical schools, altering legislation and changing roles is an effort that may take years to bring forth. Therefore, planning has to target a long enough time horizon if it is to be useful and applicable and has to be done pre-emptively.

It now seems obvious that, like any other complex problem, all the determining pieces of the system and their interdependent relationships must be duly accounted for. Therefore, pressing for integrated approaches is still a valid and up-to-date concern. Furthermore, envisioning the health worker in its entire complexity makes it possible to address the problem more comprehensively, leaving room to improvements in productivity and in the distribution of work without having to directly interfere with the training process or with the health providers. Operations research and lean management are particularly relevant in this area. This strategy may be, in fact, a first attempt to solve the lack ofprofessionals.

The results of our review point in one clear direction: accurate HHR planning requires an approach that is both integrated and flexible, featuring supply and demand (potential and effective) and incorporating less tangible factors, such as skill mix and productivity. The road to accurate HHR planning cannot abstainfrom this.

## Endnote

^a^ Henceforth, the term ’approach’ is used loosely to refer to the conceptual methodology employed rather than to the technical and scientific apparatus used to obtain a projection or forecast.

## Abbreviations

HHR: Health-care human resources
OECD: Organisation for Economic Co-operation and Development
WHO: World Health Organization
